# Molecular basis for GTP recognition by light‐activated guanylate cyclase RhGC

**DOI:** 10.1111/febs.15167

**Published:** 2019-12-20

**Authors:** Agata Butryn, Hadeeqa Raza, Heather Rada, Isabel Moraes, Raymond J. Owens, Allen M. Orville

**Affiliations:** ^1^ Diamond Light Source Limited Didcot UK; ^2^ Research Complex at Harwell Didcot UK; ^3^ Protein Production UK Research Complex at Harwell Didcot UK; ^4^ Membrane Protein Laboratory Diamond Light Source Limited Didcot UK; ^5^ Present address: National Physical Laboratory Teddington Middlesex TW11 0LW UK

**Keywords:** cGMP, cyclic GMP, guanylate cyclase, guanylyl cyclase, retinylidene photoreceptor

## Abstract

Cyclic guanosine 3′,5′‐monophosphate (cGMP) is an intracellular signalling molecule involved in many sensory and developmental processes. Synthesis of cGMP from GTP is catalysed by guanylate cyclase (GC) in a reaction analogous to cAMP formation by adenylate cyclase (AC). Although detailed structural information is available on the catalytic region of nucleotidyl cyclases (NCs) in various states, these atomic models do not provide a sufficient explanation for the substrate selectivity between GC and AC family members. Detailed structural information on the GC domain in its active conformation is largely missing, and no crystal structure of a GTP‐bound wild‐type GC domain has been published to date. Here, we describe the crystal structure of the catalytic domain of rhodopsin–GC (RhGC) from *Catenaria anguillulae* in complex with GTP at 1.7 Å resolution. Our study reveals the organization of a eukaryotic GC domain in its active conformation. We observe that the binding mode of the substrate GTP is similar to that of AC–ATP interaction, although surprisingly not all of the interactions predicted to be responsible for base recognition are present. The structure provides insights into potential mechanisms of substrate discrimination and activity regulation that may be common to all class III purine NCs.

**Database:**

Structural data are available in Protein Data Bank database under the accession number 6SIR.

**Enzymes:**

EC4.6.1.2.

## Graphical abstract

Synthesis of cGMP, an important intracellular signalling molecule, is catalysed by guanylate cyclases. Although detailed structural information is available, current data do not provide a sufficient explanation for the substrate selectivity between guanylate and adenylate cyclases. We present the crystal structure of the GTP‐bound catalytic domain of rhodopsin–GC from *Catenaria anguillulae* at 1.7 Å resolution. Our study provides insights into potential mechanisms of substrate discrimination and activity regulation.

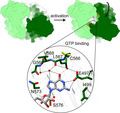

**Abbreviations**
AC: adenylate cyclase
ATP: adenosine‐5′‐triphosphate
cAMP: cyclic 3′,5′‐adenosine monophosphate
cGMP: cyclic 3′,5′‐guanosine monophosphate
GC: guanylate cyclase
GTP: guanosine‐5′‐triphosphate
NC: nucleotidyl cyclase
sGC: soluble guanylate cyclase

## Introduction

Guanylate cyclases (GCs) convert guanosine 5′‐triphosphate (GTP) into one of the most important intracellular messengers, cyclic guanosine 3′,5′‐monophosphate (cGMP). cGMP is central to many transduction pathways where it propagates signals in processes that include neurotransmission, blood pressure regulation, bone growth, lipolysis or muscle contraction 1. All GCs belong to the class III nucleotidyl cyclase (NC) family that also includes many adenylate cyclases (ACs), which perform an analogous function of converting adenosine 5′‐triphosphate (ATP) to cyclic adenosine 3′,5′‐monophosphate (cAMP). Class III ACs and GCs are widely distributed through all kingdoms of life and are closely related in primary sequence 2. They have evolved as dimeric head‐to‐tail wreath‐like assemblies with the active site(s) formed at the interface between the catalytic subunits/subdomains 3.

Mechanistic insights into the activity of class III NCs have been derived from dozens of crystal structures of AC domains in a ligand‐free form or bound to substrate‐based inhibitors, substrates or ATP analogues in various conformations 3, 4, 5, 6, 7, 8, 9, 10, 11. Structural analysis suggests that the AC dimer undergoes a functional conformational change that involves ‘closing’ of the active site region that brings the catalytic residues distributed across the subunit interface into the proper register. Such stimulatory dynamic changes can be governed *in trans* by factors directly binding to the catalytic domain such as G proteins 3 and bicarbonate 4, 7, or *in cis* in prokaryotes and lower eukaryotes 12, 13, 14.

Despite many structural insights into AC action, our understanding of mechanisms of the reaction catalysed by GCs is largely missing. In particular, the current literature does not provide a sufficient explanation for the NC substrate selectivity. Most of the GC structures available to date represent ligand‐free, atypical head‐to‐head or canonical head‐to‐tail dimers that are inactive due to misaligned active site residues 15, 16, 17, 18, 19, 20. A few reports describe crystal structures of the GC domain in the active conformation; however, they exploit enzymes of altered specificity 10, 11. Important new information has recently emerged from the 3.8 Å resolution cryo‐EM study of the human soluble GC (sGC) in its inactive and GMPCPP‐bound NO‐activated state 21, suggesting that all type III NCs undergo dynamic structural changes as part of the catalytic cycle.

An aquatic fungus, *Blastocladiella emersonii*, uses cGMP‐mediated signalling to control zoospore phototaxis 22, 23, 24, 25. *B. emersonii* genome sequencing data revealed the presence of a unique gene fusion comprising a microbial (type I) rhodopsin fused to a GC domain 26. Additionally, orthologues were identified in the genomes of related species, *Catenaria anguillulae* and *Allomyces macrogynus*, but not in other fungi 26. The rhodopsin‐GC protein fusion (hereafter referred to as RhGC) was directly demonstrated to function as a light‐activated GC that has a great potential as a new optogenetic tool linking to reversible cGMP manipulation in the cell 27, 28.

Crystal structures of the soluble GC domain of *B. emersonii* RhGC (BeGC) 20 and a calcium‐ and ATPαS‐bound dimeric form of the *C. anguillulae* RhGC GC domain with several mutations that transform substrate specificity into an AC (CaAC·ATPαS·Ca^2+^) 11 have been recently described. Here, we report the crystal structure of the wild‐type GC domain of RhGC from *C. anguillulae* in its GTP‐ and calcium‐bound form (CaGC·GTP·Ca^2+^). This is the first study that reveals the organization of a native GC domain in its substrate‐bound state. The structural analysis presented here provides important insights into substrate binding and selectivity of class III NCs.

## Results

### Structure of the CaGC·GTP·Ca^2+^ complex

**Figure 1:**
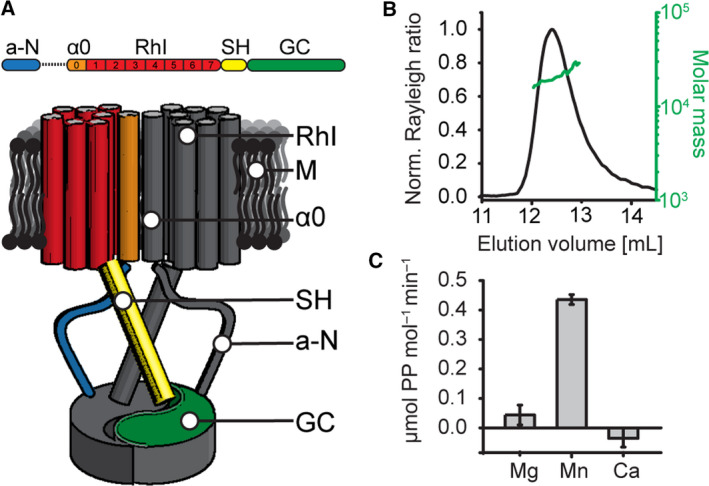
Characterization of the crystallized construct. (A) Schematic domain organization of the full‐length RhGC (top) and its potential dimeric assembly (bottom). M: membrane. From the N terminus: a‐N: N‐terminal autoinhibitory region. α0: predicted additional transmembrane helix. RhI: type I rhodopsin domain. SH: signalling helix. GC: GC domain. On the bottom panel, one of the monomers is colour‐coded, whereas the second one is shown in grey. (B) SEC‐MALS analysis of the CaGC construct. The chromatogram displays normalized Rayleigh ratio (black) and molar mass calculated by MALS (20 kDa, green). (C) Activity of the CaGC construct in solution in the presence of different divalent cations measured as concentration of generated pyrophosphate. Activity was measured in triplicates. Depicted values represent the mean ± SD.

Rhodopsin–GC (RhGC) contains an N‐terminal autoregulatory element, type I rhodopsin domain, coiled‐coli region (signalling helix) and catalytic GC domain on the C terminus (Fig. 1A). We cloned, expressed and purified the C‐terminal part of the *C. anguillulae* RhGC (residues 442–626, further denoted as CaGC) in *Escherichia coli*. Although RhGC is anticipated to act as a dimer like all other GCs 29, 30, the isolated GC domain is monomeric in solution (Fig. 1B and 20). In many of our crystallization conditions, we obtained crystals with unit cells comprising monomeric or unconventional head‐to‐head disulphide bond‐stabilized dimeric assemblies that have been reported by others 11, 20; these will not be discussed further.

In both ACs and GCs, magnesium is the physiological active site ion 5, 31. Despite its monomeric nature in solution, our isolated CaGC was active in the presence of magnesium or manganese (Fig. 1C), confirming previous results 11, 20. As observed in other GCs, CaGC was inactive when calcium was used 16. This led us to postulate that the addition of GTP and calcium might promote the stabilization of the active dimer for use in crystallization trials. Indeed, in the presence of GTP and calcium chloride, we obtained distinct orthorhombic crystals, which did not grow in the absence of these additives. These crystals diffracted to 1.7 Å resolution, and we solved the structure of CaGC·GTP·Ca^2+^ by molecular replacement as described further below. The atomic model was refined to *R*_work_/*R*_free_ of 18.2/21.1% with good statistics with two dimers in the asymmetric unit (Table 1).

**Table 1:** Data collection and refinement statistics. Statistics for the highest‐resolution shell are shown in parentheses

	CaGC·GTP·Ca^2+^, PDB 6SIR
Data	
Space group	*P*2_1_2_1_2_1_
Unit cell *a, b, c,* α, β, γ (Å, º)	58.65, 95.17, 138.66, 90, 90, 90
Resolution range (Å)	36.30–1.70 (1.76–1.70)
Completeness (%)	98.8 (97.8)
Total reflections	1 121 287 (59 894)
Unique reflections	85 087 (8312)
Multiplicity	13.2 (13.5)
Mean *I*/sigma(*I*)	20.3 (2.2)
*R*‐merge	0.073 (1.27)
*R*‐meas	0.076 (1.32)
*R*‐pim	0.021 (0.36)
CC1/2	1 (0.79)
Refinement	
Resolution range (Å)	36.30–1.70 (1.76–1.70)
No. reflections (work)	85 085 (8311)
No. reflections (free)	4316 (415)
*R*‐work	0.182 (0.273)
*R*‐free	0.211 (0.303)
No. of non‐H atoms	6546
Protein	5809
Ligand	162
Solvent	575
R.m.s. deviations	
Bonds (Å)	0.007
Angles (°)	1.06
Average *B* factors (Å^2^)	28.9
Protein	27.9
Ligand	34.7
Solvent	37.5
Ramachandran plot	
Favoured regions (%)	98.2
Additionally allowed (%)	1.8
Outliers (%)	0.0

**Figure 2:**
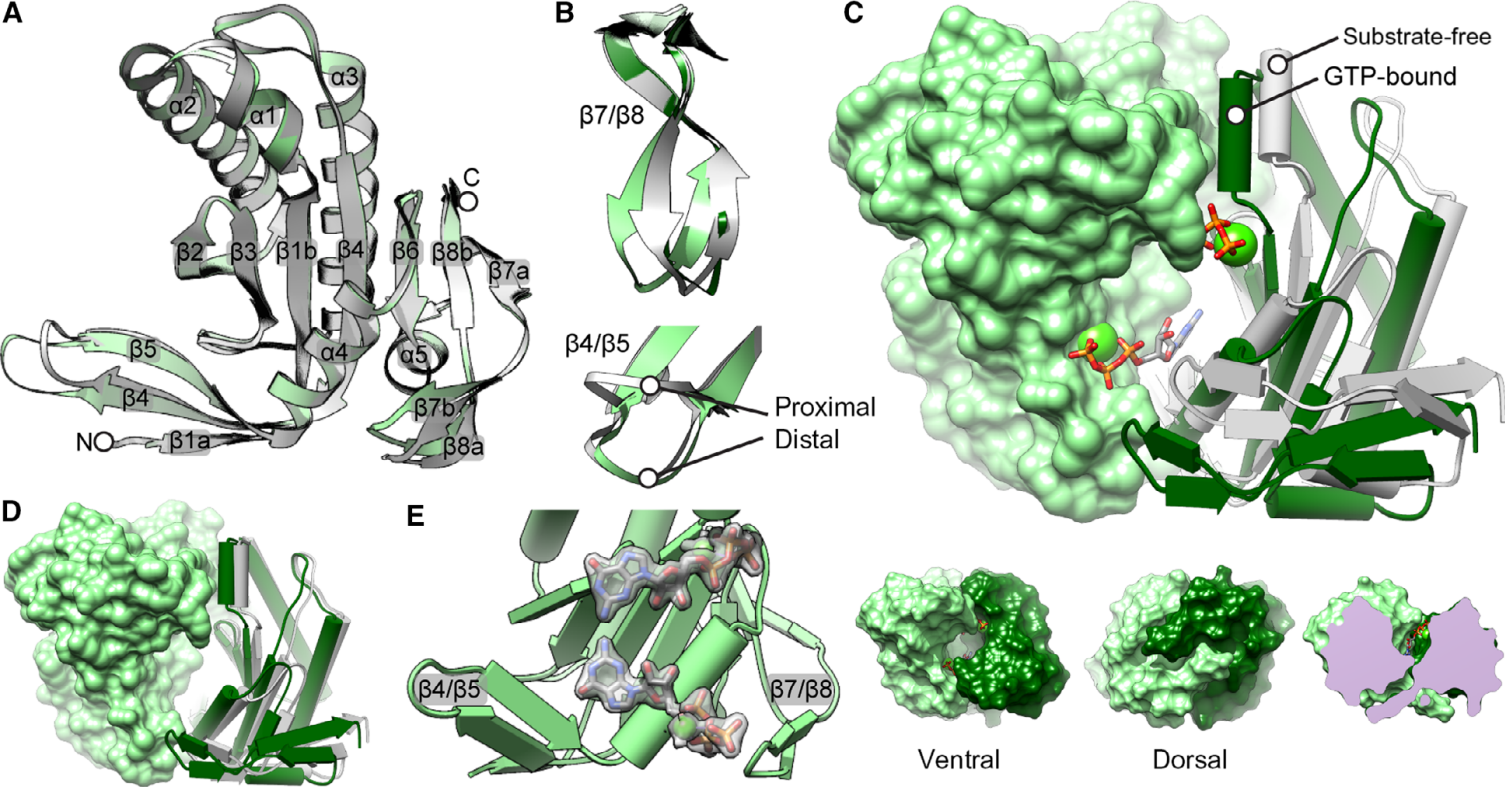
CaGC·GTP·Ca^2+^ structure overview. (A) Cartoon representation of the CaGC (light green, PDB 6SIR) and CaAC monomer (grey, PDB 5OYH 11). Secondary structure elements are depicted and labelled β1–β8 for the strands and α1–α5 for the helices. N and C terminus are labelled N and C, respectively. (B) Comparison of the tongue regions in CaGC and CaAC. Top: conformation of the β7/β8 tongues differs between CaGC monomers (light and dark green) whereas it is same for all CaAC monomers (grey). Grey and dark green conformations are almost perfectly overlapping. Bottom: conformation of the β4/β5 tongues differs between CaAC monomers within each dimer (both grey), whereas it is distal for all CaAC monomers (light green). (C) Top: comparison of the CaGC dimer (light and dark green) and its ligand‐free form modelled based on the inactive sGC dimer (PDB 6JT1 21). View from the ventral side. Structures were superimposed via the light green monomer shown as single surface. Calcium ions are depicted as green spheres. GTP molecules are shown as sticks. Bottom: CaGC·GTP·Ca^2+^ dimer along its twofold axis viewed from the ventral (left) and dorsal side (middle) represented as solvent accessible surface. Right bottom panel shows cross section of the dimer with the view on one of the substrate binding pockets. (D) Comparison of CaGC·GTP·Ca^2+^ dimer and its model based on the sGC·GMPCPP·2×Mg^2+^ assembly (PDB 6JT2 21). Structures were superimposed via the light green monomers shown as single surface. Second subunit in CaGC·GTP·Ca^2+^ (dark green cartoon) is in almost identical relative orientation as in sGC·GMPCPP·2×Mg^2+^ (grey cartoon). (E) 2*F*_o_–*F*_c_ electron density map carved within 2 Å distance around GTP and calcium, displayed at 1.5 σ level. For clarity, second monomer was omitted. Molecular graphics and analyses were performed with the ucsf chimera package 52.

The crystal structure of the *C. anguillulae* RhGC GC domain engineered with mutations into an AC (CaAC) and bound to ATPαS has been described previously 11. Primary sequences of CaAC and CaGC differ only in two positions (E153K/C566D), which are critical for substrate selectivity 11. The atomic models of CaGC monomer presented herein and CaAC are almost identical (RMSD of 0.3–0.9 Å across all atoms, Fig. 2A) with the largest differences found only in the ‘tongue’ regions. In CaAC·ATPαS·Ca^2+^, all β7/β8 tongues are similar and are located on the ventral side of the dimer, whereas β4/β5 tongues (dorsal side) are found in two different conformations in the dimer (distal and proximal, Fig. 2B). On the contrary, β7/β8 tongues of CaGC monomers are somewhat less well defined and the electron density of chains A and C suggests the presence of two coexisting conformations from which only the more prominent was modelled. Unlike in CaAC·ATPαS·Ca^2+^, in CaGC·GTP·Ca^2+^ β4/β5 tongues are entirely uniform and all adopt the distal conformation. Although in both structures conformations of β tongues are affected by the crystal packing, these differences might suggest that these regions play a functional role (see Discussion).

The CaGC·GTP·Ca^2+^ atomic model presented here represents the ‘active’ state of the wreath‐like type III NC assembly. The monomers are related to each other by a translocation and rotation around the centre of the dimer interface in comparison with an inactive ligand‐free form of GC (Fig. 2C 21). Despite some differences, the CaGC·GTP·Ca^2+^ dimer conformation is almost identical as in CaAC·ATPαS·Ca^2+^ (RMSD between the dimer conformations ~ 0.4 Å) and in the reconstruction of the activated human sGC·GMPCPP·2×Mg^2+^ complex (RMSD 1.4 Å, Fig. 2D) 21. The differences account for the slight relative tilt of the subunits around the axis parallel to the dimer interface. The ‘closed’ conformation was likely induced by the presence of two electron‐rich atoms (tentatively assigned as calcium) and two GTP molecules that occupy the active sites located at the interface between the monomers (Fig. 2E, discussed further below). GTP and calcium binding induces closure of the ventral side of the dimer and overall decrease in the interface area between the subunits from predicted 1600 to 1300 Å^2^, as calculated by PISA 32.

### CaGC·GTP·Ca^2+^ active site

**Figure 3:**
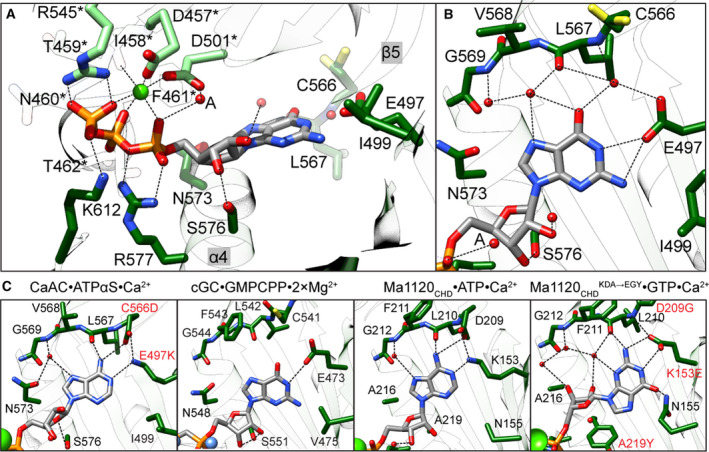
Active site of CaGC·GTP·Ca^2+^ complex. (A) Active site in CaGC·GTP·Ca^2+^ structure. Chains A and B are in light and dark green, respectively. The calcium ion is shown as a green sphere. The GTP, residues crucial for substrate binding and turnover are shown as sticks. Water molecules are shown as red spheres. The metal coordination and selected hydrogen bonds between protein, triphosphate and ribose are shown as black dashed lines. (B) Close‐up view of the protein–guanine base interaction in the CaGC·GTP·Ca^2+^ structure. (C) From left to right: close‐up view of the protein–base interaction in CaGC·ATPαS·Ca^2+^ (PDB 5OYH 11, sGC·GMPCPP·2×Mg^2+^ (PDB 6JT2 21), Ma1120_CHD_·ATP·Ca^2+^ (PDB 5D15 10 and Ma1120_CHD_ ^(^ ^KDA^ ^→^ ^EGY^ ^)^ ·GTP·Ca^2+^ (PDB 5D0G 10) structures. Introduced mutations are marked in red. In all panels, maximum acceptor–donor distance is 3.5 Å and colour coding as in A. Molecular graphics and analyses were performed with the ucsf chimera package 52.

**Figure 4:**
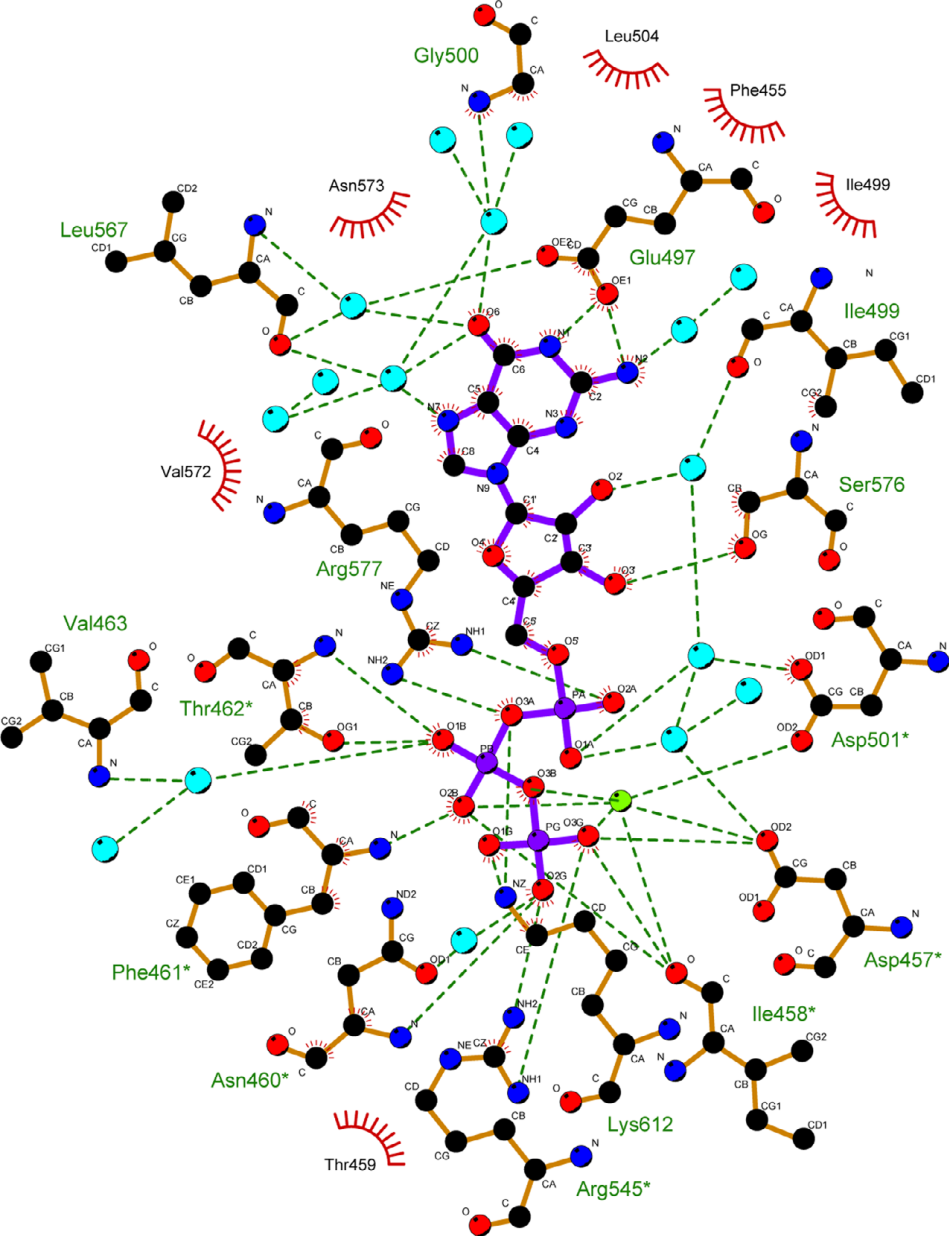
GTP–CaGC interaction in one of the active sites. Residues involved in hydrophobic contacts are shown as a curved comb. Ionic and hydrogen bond interactions (maximal donor–acceptor distance = 3.5 Å) are shown as dark green dotted lines. Calcium and waters are represented by light green and light blue spheres, respectively. Figure generated using ligplot+ 53.

This CaGC·GTP·Ca^2+^ structure is the first to visualize a wild‐type GC domain dimer bound to its natural substrate. The phosphate groups of GTP are coordinated by the backbone of Asn460*, Phe461*, Thr462* and side chain of Arg545*, which is believed to aid the exit of the product pyrophosphate (residues from the monomer that binds the triphosphate tail are marked with an asterisk, Figs 3A and 4). The other subunit chain contributes two residues to the active site. Lys612 interacts with the GTP γ phosphate, and side chain of Arg577 is positioned appropriately to help stabilize a transition state in the reaction cycle 5, 33. The density of these two side chains is less well defined in chains A and C that display two conformations of the β7/β8 tongue.

Two divalent cations, typically magnesium or manganese, are postulated to facilitate substrate binding (ion B) and catalytic turnover (ion A) 5, 30. With a few exceptions, in ligand‐ and calcium‐bound NC structures, only the high‐affinity ion B site is occupied 4, 6, 7, 8, 10, 11, 21, 34, 35. This is most probably due to the significantly larger atomic radius of calcium compared to magnesium or manganese. In our CaGC·GTP·Ca^2+^ model, calcium also only binds in the position analogous to ion B and is coordinated by a pair of aspartic acids Asp457* and Asp501*, α‐, β‐ and γ‐phosphate, as well as the backbone carbonyl of Ile458*. Our electron density maps are most consistent with a water molecule occupying the ion A position, which explains our observation that calcium does not support catalytic activity. Despite the absence of low‐affinity catalytical ion A, the formation of NC dimers in its fully active conformation is well supported 4, 11, 36.

In contrast to the polyphosphate tail, in most of the NC domain structures ribose moiety makes no or only a few direct contacts with the protein. These contacts vary among NC structures and are likely to change in the presence of the transiently bound A site metal during the catalysis. The conserved Asn and Ser (α4) residues were implicated as a catalytically important whereby they help to orient the sugar in the active site of ACs by lowering the activation energy of the transition state 5, 7, 33, 37. Neither in our CaGC·GTP·Ca^2+^ model, nor in CaAC·ATPαS·Ca^2+^ or sGC·GMPCPP·2×Mg^2+^ structures does the sugar moiety form hydrogen bonds to topologically equivalent Asn (Fig. 3B). However, a hydrogen bond is formed between the Ser residue via ribose O3′ in CaGC·GTP·Ca^2+^, via O2′/O3′ in sGC·GMPCPP·2×Mg^2+^ or via O4′ in CaAC·ATPαS·Ca^2+^ (Fig. 3C).

**Figure 5:**
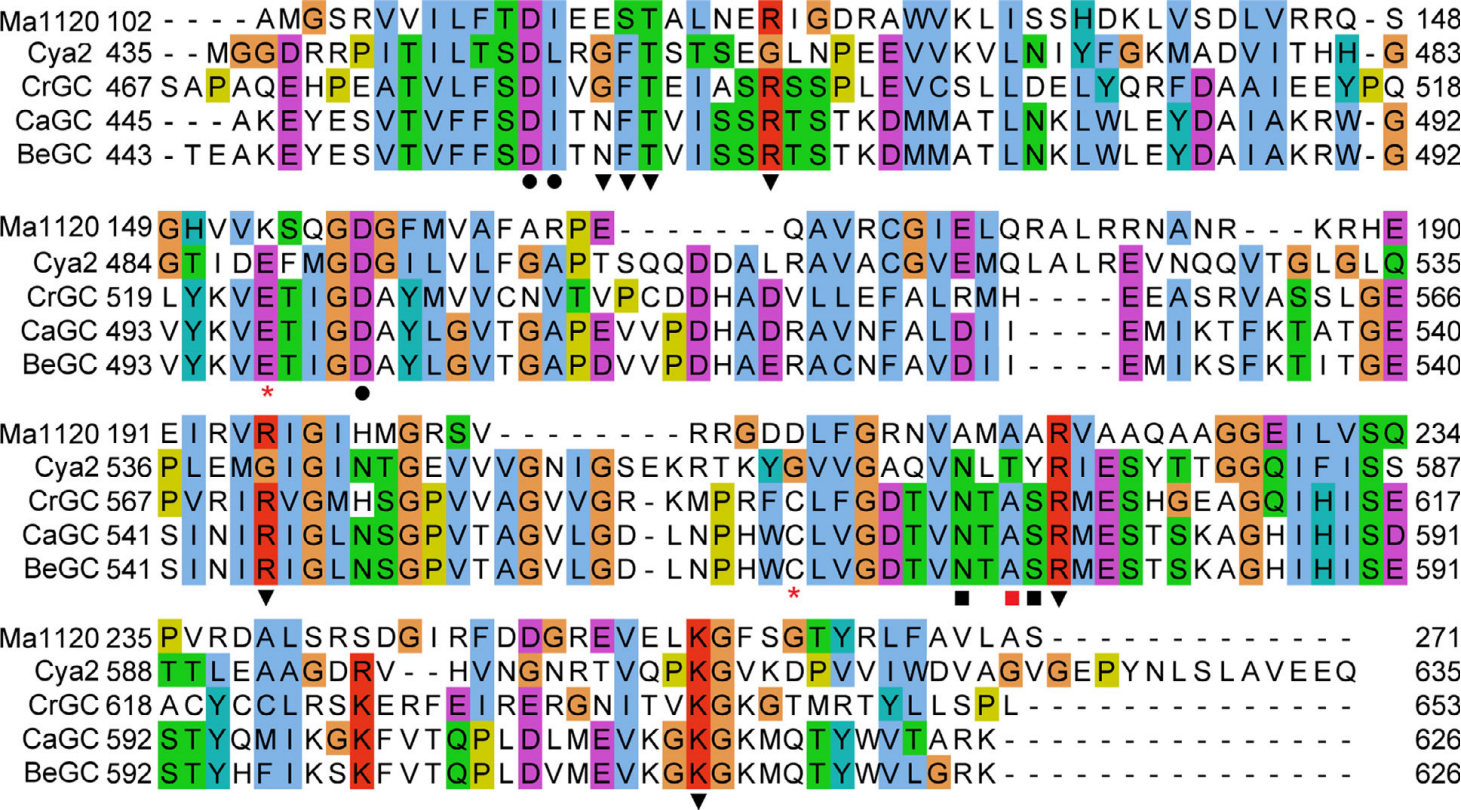
Alignment of sequences of cyclases: *Catenaria anguillulae* CaGC (PDB 6SIR, this work), *Blastocladiella emersonii* BeGC (PBD 6AOB 20), *Mycobacterium avium* Ma1120 (PDB 5D15 10), *Synechocystis* sp. Cya2 (PDB 2W01 16), *Chlamydomonas reinhardtii* GC (PDB 3ET6 15). Amino acids that coordinate to the catalytical ion (●), phosphate (▼), ribose (■) or base (*) are marked. Positions marked with red (*) are mutated in CaAC. Positions marked with red (*) and with red (■) are mutated in Ma1120_CHD_ ^(^ ^KDA^ ^→^ ^EGY^ ^)^ mutant. Alignment was created using clustal omega 54 and jalview 55.

In NCs, the purine ring binds in a hydrophobic pocket within the active site with few polar interactions that serve to discriminate between ATP and GTP. Catalytic specificity for ATP in ACs is predominantly determined by two residues: Lys and Asp (or Thr), which have been shown to directly interact with N1 and N6 atoms of adenine base, respectively 5. A few of the AC structures contain unmodified ATP 6, 8, 10, 14. Most of the ligand‐bound ACs were cocrystallized in the presence of a substrate analogue. They provide useful information, but also have significant limitations, for example, often contain substrate modifications that eliminate interactions critical to catalysis 4, 5, 33. The *Mycobacterium avium* Ma1120_CHD_ 10 and *Arthrospira platensis* CyaC 4 structures are good examples of wild‐type ACs crystallized with the ligand in a conformation that represents a state well aligned for the nucleophilic attack and in which both direct interactions between the specificity‐determining residues and substrate are present. In GCs, Lys and Asp present in ACs are replaced by Glu and Cys (or Ser) that are believed to mediate recognition of the exocyclic amine and carbonyl group of the guanine, respectively 38. The second guanine‐specifying residue shows lower conservation indicating that its interaction varies in different members of the GC family 16 (Fig. 5). In BeGC and CaGC, residues Glu497 and Cys566 control substrate specificity 11, 39. As expected, the guanine in the CaGC·GTP·Ca^2+^ complex is positioned as GMPCPP in the active site of sGC and this orientation is equivalent to adenine in most of the AC structures. In particular, Glu497 appears to form a hydrogen bond to N1/N2 of guanine. However, in all monomers Cys566 side chains clearly adopt rotamer conformations that face away from the base and are inconsistent with hydrogen bond formation (Fig. 3B). Instead, the guanine moiety is indirectly stabilized by water molecules that also interact with backbone atoms of Leu567 and Gly500. Although the 3.8 Å resolution of the sGC·GMPCPP·2×Mg^2+^ cryo‐EM model limits the accuracy of the substrate–enzyme interactions, it does provide insight into GTP recognition 21. For example, Glu473 is also well positioned to form a hydrogen bond to the guanine N1 or N2 atoms. The Cys541 residue interaction with the base is less obvious because it faces away from the active site; nevertheless, it could form a hydrogen bond with the carbonyl group if it assumed a different rotamer (Fig. 3C). The lack of the second canonical interaction was also reported in the structural analysis of the dual‐specificity triple mutant of Ma1120_CHD_, Ma1120_CHD_^(KDA→EGY)^, in which the base is flipped by 180° and the observed mode of interaction is different than that predicted for GCs.

## Discussion

**Figure 6:**
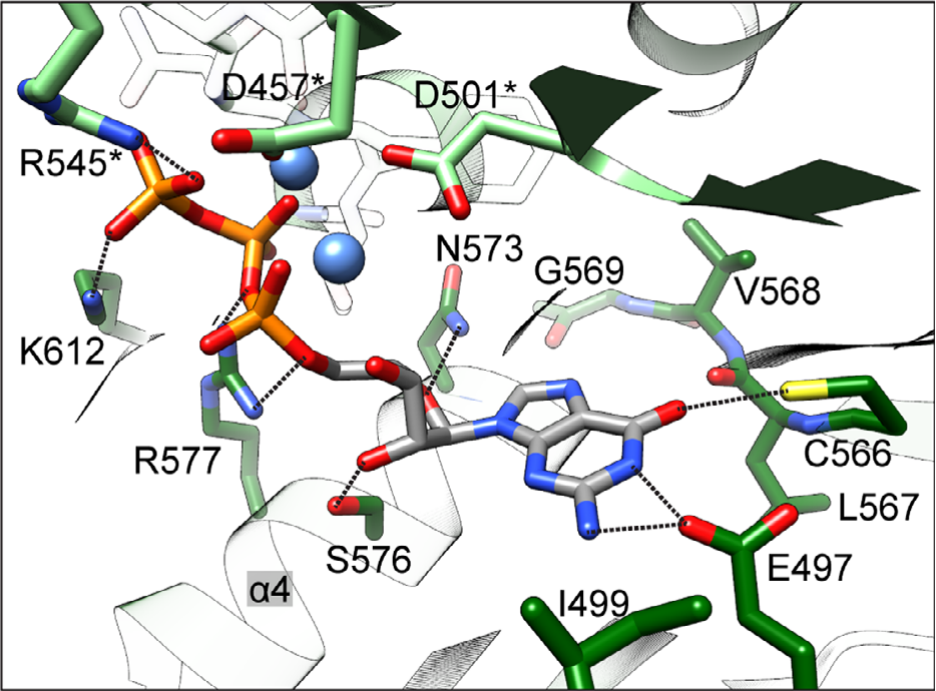
Hypothetical model of the CaGC active site in the presence of two catalytic Mg^2+^ cations. Model was based on the Ma1120_CHD_·ATP·Ca^2+^ crystal structure (PDB 5D15) 10. Magnesium ions are shown as blue spheres. Selected hydrogen bonds between the protein and GTP are shown as black dashed lines. Colour coding is as in Fig. 3. Molecular graphics and analyses were performed with the ucsf chimera package 52.

Despite progress in our knowledge of the structure and regulation of ACs, the molecular mechanisms that determine GC substrate specificity and control its activity are still poorly understood. Recent cryo‐EM structures of the human sGC provided important new insights into the mode of enzyme–substrate interaction of a wild‐type GC 21. In our work, we extend the interpretation of the cryo‐EM results by providing a high‐resolution crystal structure for a GTP complex in a eukaryotic light‐activated GC, RhGC. Together, these results support the conclusion that GTP binding and recognition by GCs are very similar to classical AC–ATP interaction(s). This explains why the specificity of type III NCs is achieved not upon substrate binding, but rather during catalysis 8, 16, 40. Surprisingly, in our CaGC·GTP·Ca^2+^ structure only one canonical, guanine‐specific hydrogen bond is formed via Glu497 located on β5. Therefore, our data support the hypothesis that, although in ACs both specificity‐determining residues seem to form direct hydrogen bonds with ATP, in GCs Glu–N1/N2 interaction plays the major role in GTP specificity. The second base‐specific residue could serve an alternative function; rather than forming a hydrogen bond to guanine, it could provide shape complementarity with the nucleotide base 10, 16. The organization of the active site appears to be sensitive to local rearrangements. Therefore, the Cys566–base interaction(s) could impact regulation of protein activity. This is reinforced by the observation that this residue is not located within the subunit chain of the (pseudo)dimer to which GTP triphosphates are tightly anchored via strong protein and metal ion interactions with highly conserved residues 30. It is also very likely that perturbations in the active site geometry originate from the presence of noncatalytic ions or substrate modifications. For example, in many AC structures of nonproductive enzyme–substrate complexes, one or even both base interactions are missing 6, 7, 8, 34, 35. In the CaGC·GTP·Ca^2+^ structure, the catalytic ion (site A) is unoccupied. Because our complex is inactive, our atomic model does not represent a reaction‐competent state. We speculate that the coordination mode we observe, wherein the guanine–Cys566 hydrogen bond formation is hindered by the presence of calcium, is the principal reason our complex does not support catalysis. Nevertheless, hypothetical modelling of the CaGC active site suggests that all canonical interactions could be supported in the presence of two catalytic ions (Fig. 6).

RhGC activity is regulated by light via the type I rhodopsin domain located towards the N‐terminal region the GC domain. The question remains open as to how light induces structural changes in the rhodopsin sensor and how these are transmitted to the catalytic domain. A parallel coiled‐coil element formed by two signalling (S)‐helices is a distinct integral element involved in the dimerization of diverse protein fusions 41. In RhGC, such an element is thought to bridge the rhodopsin and GC domain. In addition to playing a structural role, the S‐helix has also been proposed to be involved in the signal transduction from the N‐terminal sensory to various C‐terminal catalytic domains, including type III NCs 19, 21, 41, 42, 43. According to this model, the activation signal propagates through the S‐helix and a short ‘handle’ helix (also described as cyclase transducer element) towards the catalytic domain. We favour the idea that the enzyme catalysis is modulated through the changes in the N‐terminal dimer interface, which subsequently affect the conformation of β4/β5 tongues and thereby affect the active site region where the base discrimination takes place 11, 14, 42. Although a primary signal transduction mechanism may be common to type III NCs, the distinct regulatory mechanisms are likely to be protein‐specific. For example, in the photoactivatable AC from *Beggiatoa* sp. (bPAC), the isolated catalytic domain is inactive and light stimulation promotes a conformational change to a catalytically competent state of the full‐length protein 14. Unlike bPAC, isolated GC domain of RhGC is constitutively active. This observation suggests that in RhGC the light signal might release the constraints on an otherwise intrinsically active enzyme 11, 20. Apart from the cyclase transducer element, the GC domain interacts with other regions of the protein through distinct interfaces providing an additional level of control. This includes conserved N terminus that precedes the type I rhodopsin domain 11, 27. Truncations of this part of the protein, which is unique to RhGC, either disrupt light‐dependent regulation of activity 27, 28 or turn the protein entirely inactive 39. Clearly, further studies on the full‐length RhGC and other NCs in various physiological states will be required to understand how the interplay between different interactions is responsible for the regulation of the catalytic activity and how it differs between ACs and GCs.

## Materials and methods

### Cloning and protein purification

DNA encoding *C. anguillulae* RhGC was obtained from Integrated DNA Technologies (Leuven, Belgium) as a synthetic gene with a codon distribution optimized for expression in *E. coli*. The DNA construct corresponding to the catalytic domain (amino acid residues 442–626) was PCR‐amplified and cloned into pOPINM vector 44 with an N‐terminal His_6_‐MBP fusion and HRV3C protease cleavage site. His_6_‐MBP‐CaGC was transformed into *E. coli* Lemo21(DE3) cells (New England Biolabs, Hitchin, UK) and grown in TB medium (Merck Millipore, Watford, UK) at 37 °C until OD_600 nm_ reached 1.0. Expression was induced by the addition of IPTG at 1 mm final concentration and carried out at 18 °C overnight. After cell harvesting, the pellet was resuspended in lysis buffer (50 mm Tris 7.5, 500 mm NaCl, 10 mm imidazole, 2 mm β‐mercaptoethanol) and sonicated for 15 min on ice. After centrifugation, supernatant was loaded onto HisTrap HP column (GE Healthcare, Little Chalfont, UK) equilibrated in the lysis buffer. The bound fraction was washed extensively with lysis buffer and eluted with elution buffer (50 mm Tris 7.5, 500 mm NaCl, 250 mm imidazole, 2 mm β‐mercaptoethanol). HRV3C protease was added to the elution fractions to cleave off the N‐terminal tag. Sample was then dialysed overnight against 20 mm Tris pH 7.5, 50 mm NaCl, 2 mm β‐mercaptoethanol and loaded onto HiTrap Q HP column (GE Healthcare). Flow‐through fractions were then concentrated and loaded onto HiLoad 16/600 Superdex 75 pg column (GE Healthcare) equilibrated in 5 mm Tris pH 7.8, 50 mm NaCl, 2 mm β‐mercaptoethanol. Protein fractions were pooled and concentrated to 20 mg·mL^−1^, flash‐frozen in liquid nitrogen and stored at −80 °C.

### SEC‐MALS

Size exclusion chromatography coupled with multiangle light scattering (SEC‐MALS) was performed on an Agilent1100 system at 22 °C. Hundred microlitres of the protein solution at 5 mg·mL^−1^ was injected onto Superdex 75 10/300 Increase column connected to ÄKTA pure (GE Healthcare) equilibrated in 50 mm TRIS pH 7.8, 50 mm NaCl, 0.5 mm TCEP and run at 1.3 mL·min^−1^. The chromatography system was connected to a multiangle light scattering detector DAWN HELEOS‐II (Wyatt Technology, Haverhill, UK) and Optilab T‐rEX refractometer (Wyatt Technology) connected in‐line. Data acquisition and processing were performed using astra software (Wyatt Technology).

### Pyrophosphate assay

To quantitate the level of generated cGMP, Phosphate Assay Kit (MAK168, Sigma‐Aldrich, Gillingham, UK) was used to determine the amount of pyrophosphate. 4.7 μm CaGC was mixed with 0.8 mm GTP in 50 mm HEPES 7.5, 100 mm NaCl and 2.7 mm MgCl_2_/MnCl_2_/CaCl_2_. After 1 h of incubation at 22 °C, the reaction was terminated by adding Master Reaction Mix, followed by 20‐min incubation at 22 °C. Subsequently, fluorescence measurement (excitation 316 nm, emission 456 nm) was taken on a Spectramax M5 plate reader (Molecular Devices, Wokingham, UK). All measurements were done in triplicates.

### Protein crystallization

Crystallization trials were done using CaGC at 20 mg·mL^−1^ premixed with GTP and CaCl_2_ (both at 1 mm final concentration). Several screening conditions yield protein crystals, most of which contained only protein and revealed no substrate bound and/or crystals of the protein–substrate complex that diffracted poorly. Well‐diffracting crystals of the CaGC·GTP·Ca^2+^ complex in an orthorhombic space group were grown in 0.1 m MIB pH 4.4 and 22–24% PEG 2000 at 20 °C. Crystals appeared after one or two days as 300 μm × 20 μm × 20 μm rods. Crystals grew exclusively in the presence of GTP and CaCl_2_. For data collection, crystals were cryo‐cooled in mother liquor supplemented with 25% glycerol by plunging rapidly into liquid nitrogen.

### Data collection and processing

Data were collected at I24, Diamond Light Source, UK, from a single crystal held at 100 K. Indexing and integration were done in DIALS to 1.7 Å resolution in space group *P*2_1_2_1_2_1_ 45. Intensities were scaled and merged in aimless 46, 47. The structure was solved by molecular replacement using phaser 48 with the cyanobacterial GC Cya2 monomer as the search model (PDB 2W01) 16. Distinctive electron density peaks in the 2*F*_o_–*F*_c_ and *F*_o_–*F*_c_ maps that corresponded to one GTP and one Ca^2+^ ion in each active site of the CaGC·GTP·Ca^2+^ complex were fitted with these ligands and included in the structure refinement. Structure was refined using phenix 49 and coot 50 and using the PDB_REDO server 51 to further optimize the model.

## Conflict of interest

The authors declare no conflict of interest.

## Author contributions

AB, IM, RJO and AMO planned research; AB, HRaz and HRad performed experiments; AB and AMO analysed data and wrote the paper; and all authors commented on the final manuscript.
